# Enhancing Patient‐Centered Communication in Hemodialysis Symptom Management Care‐Development and Validation of the HSB‐HD Scale for Assessing Help‐Seeking Behavior in Hemodialysis Patients: A Multiphase Cross‐Sectional Study

**DOI:** 10.1155/jonm/8834451

**Published:** 2026-02-27

**Authors:** Xutong Zheng, Aiping Wang

**Affiliations:** ^1^ Department of Public Service, The First Affiliated Hospital of China Medical University, Shenyang, Liaoning, China, cmu.edu.cn

**Keywords:** classical test theory, exploratory factor analysis, help-seeking behavior, hemodialysis, item response theory, psychometric evaluation, scale development

## Abstract

**Background:**

Chronic kidney disease (CKD) is a global health challenge, and hemodialysis is a common treatment for end‐stage renal disease. Patients undergoing hemodialysis often face significant symptom burden, affecting their quality of life. Timely help‐seeking behavior (HSB) is crucial for initiating patient‐centered communication and effective symptom management. Yet, the specific behavioral patterns and barriers to help‐seeking in this group remain poorly understood. However, tools to assess HSB in this population are lacking.

**Methods:**

This multiphase cross‐sectional study was conducted across three hospitals in China from August to October 2024, involving 425 participants. The study developed the HSB for hemodialysis symptoms (HSB‐HD) scale based on literature reviews, expert consultations, and patient interviews. Psychometric evaluation was performed using classical test theory (CTT) and item response theory (IRT), focusing on reliability, validity (content, construct, criterion‐related, discriminant, and convergent), and measurement invariance.

**Results:**

The exploratory factor analysis (EFA) revealed a four‐factor structure (symptom detection, symptom interpretation, decision‐making for help‐seeking, and timely disclosure and action), explaining 72.9% of variance. Confirmatory factor analysis (CFA) showed a good model fit (CFI = 0.964, RMSEA = 0.045). Cronbach’s *α* was 0.953, indicating excellent internal consistency. Validity tests showed significant correlations with the EQ‐5D‐5L pain, anxiety, and VAS scores. Measurement invariance was confirmed across gender and age groups.

**Conclusions:**

The HSB‐HD scale is a reliable and valid tool for assessing HSB in hemodialysis patients. It offers a patient‐centered approach to symptom management, providing health care providers with a means to identify those who may benefit from targeted interventions, thereby improving care and quality of life.

## 1. Introduction

With the acceleration of the aging process and the increasing incidence of chronic diseases such as metabolic syndrome, the number of cases of chronic kidney disease (CKD) has been rising annually, becoming a global health issue. The global burden of disease (GBD) study indicates that CKD has become one of the leading causes of death worldwide [1]. The GBD report shows that CKD’s rank on the list of causes of death has been continuously rising, ranking 13th in 2016, 12th in 2017, and is projected to become the fifth leading cause of death by 2040 [2, 3]. The United Nations Sustainable Development Goals aim to reduce premature mortality from noncommunicable diseases by one‐third by 2030 [4]. Given the high mortality rate of CKD, addressing this disease is a crucial factor in achieving these goals. Strengthening the attention to and treatment of CKD should be prioritized.

Compared to the treatment of other chronic diseases, hemodialysis imposes an disproportionately high economic burden [5–7]. Studies have shown that in addition to the direct economic burden caused by dialysis and treatment, hemodialysis patients also experience indirect economic losses because of factors such as absenteeism from work, disability, or premature death [8]. Although it is not possible to directly reduce medical expenditure related to dialysis, it is possible to alleviate the economic burden on society and families by promoting patient rehabilitation and reintegration into society.

Maintenance hemodialysis patients experience a diverse and complex symptom phenotype, including pain, fatigue, and pruritus, stemming from the accumulation of uremic toxins and the dialysis procedure itself [9–11]. Studies indicate that a higher symptom burden is significantly associated with reduced health‐related quality of life (HRQOL) and increased mortality [12–15]. Consequently, international guidelines, such as the KDIGO consensus, identify symptom management as a priority for improving patient outcomes [16–19]. In spite of this, current care models often rely heavily on clinician‐led standardized assessments, frequently neglecting the patient’s perspective and the necessity of active symptom disclosure [20, 21]. To bridge the gap between clinical requirements and patient needs, a shift toward patient‐centered symptom management is essential.

The appearance or change of symptoms is a significant indicator of changes in a patient’s condition. Because of the complex pathophysiology of end‐stage renal disease, clinical laboratory parameters alone may lag behind the patient’s actual physiological state. Self‐reported symptoms often serve as the earliest “warning signs” of potential complications, such as fluid overload or intradialytic hypotension [22–24]. Recognizing these subjective experiences early allows nurses to implement timely interventions—such as adjusting dialysis prescriptions or optimizing medication management—thereby preventing symptom exacerbation and adverse events. This proactive approach shifts care from reactive crisis management to preventative support, which is fundamental to maintaining the patient’s functional status and improving their HRQOL [22, 25–27]. Help‐seeking behavior (HSB) is a problem‐based planned behavior associated with intentional interactions with health care professionals [28]. In the context of symptom management, HSB serves as the fundamental vehicle for patient‐centered communication, bridging the gap between the patient’s subjective experience and the nurse’s provision of care. It is considered a complex process that begins when an individual detects bodily symptoms and perceives that the symptoms may exceed their control and require professional help [29]. Current research on HSB primarily focuses on areas such as cancer, intimate partner violence, and mental health disorders [30–32]. There is limited research in the context of CKD and hemodialysis patients. Previous studies have found that hemodialysis patients often conceal their need for symptom management because of fear, lack of knowledge, low health literacy, and other factors [33]. Dialysis patients tend to refrain from communicating their symptoms to health care providers and instead adopt ineffective self‐management strategies [34, 35]. This significantly undermines patient‐centered symptom management. Therefore, it is essential to enhance the HSB in hemodialysis patients. Evaluating HSB is a prerequisite for intervention. To date, there is no available tool to assess HSB in hemodialysis patients. Thus, this study aims to develop an HSB scale for hemodialysis patients and evaluate its psychometric properties, providing a reliable assessment tool for future evaluation of HSB in these patients.

## 2. Theoretical Frameworks: Conceptualization, Derivation, Integration, and Operationalization

**FIGURE 1:**
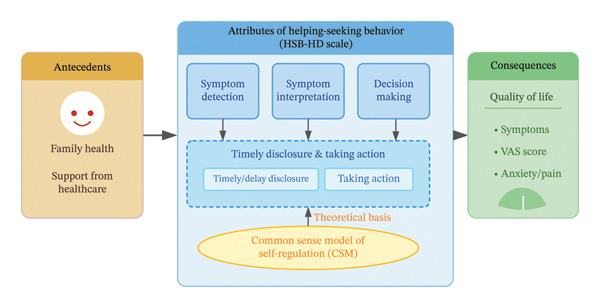
Conceptual model of helping‐seeking behavior.

The HSB‐HD is based on Maryam Momeni’s conceptual model of HSB for symptoms [36]. Momeni’s framework identifies key attributes, such as symptom detection, interpretation, and the decision‐making process for help‐seeking. However, during factor analysis, the dimensions “Taking Action for Help‐Seeking” and “Timely/Delay Disclosure” merged, indicating they are closely related in practice. This prompted the integration of the common sense model (CSM) of self‐regulation to interpret the findings [37, 38]. The CSM emphasizes that individuals’ responses to health threats—such as deciding when to seek help or disclose symptoms—are guided by their illness representations and coping strategies. These behaviors are interconnected, with the decision to disclose symptoms and take action for help‐seeking both driven by cognitive and emotional processes. Specifically, the CSM posits that both “disclosing symptoms” and “taking action” function as cohesive coping procedures activated by the patient’s illness representations. For instance, emotional representations (such as fear of burdening the medical staff) and cognitive representations (such as beliefs about symptom consequences) simultaneously influence the willingness to speak up and the physical act of seeking help. Thus, these behaviors share a common psychological pathway, explaining why they statistically converged into a single dimension in our analysis. The initial framework, based on Momeni’s model, was adapted to incorporate the CSM, which provided a more comprehensive understanding of the cognitive and emotional factors influencing HSB (see Figure 1). This adaptation clarified the overlap between “Taking Action for Help‐Seeking” and “Timely/Delay Disclosure,” justifying their merger in the factor analysis (see Table 1).

**TABLE 1:** Operationalization and conceptualization of the concept.

Domain of the concept	Conceptualization of indicators
Symptom detection	The ability to recognize and monitor physical or psychological changes during or after hemodialysis sessions.
Symptom interpretation	The cognitive process of evaluating symptom severity, attributing causes, and determining medical relevance.
Decision‐making for help‐seeking	The judgment process of deciding whether and when to seek professional assistance based on symptom evaluation.
Timely disclosure and taking action	The behavioral execution of communicating symptoms to health care providers and implementing help‐seeking strategies.

Complementing the operational definitions in Table 1, these constructs manifest in unique ways within the hemodialysis context. “Symptom Detection” refers not just to noticing changes but to the patient’s challenge of distinguishing pathological signs (e.g., intradialytic hypotension) from the “normal” baseline fatigue of dialysis therapy. “Symptom Interpretation” is heavily influenced by the patient’s chronic illness beliefs; for instance, patients may misattribute fluid overload symptoms to dietary indiscretions rather than medical urgency. “Decision‐Making” often involves a trade‐off where patients weigh the benefit of relief against the “treatment burden” of potentially extending their dialysis session. Finally, “Timely Disclosure and Action” is shaped by the social environment of the dialysis unit, where patients must navigate the fear of being perceived as “complaining” to effectively voice their needs to the nursing staff.

In this study, we operationalize the HSB‐HD as a reflective model, where the observed indicators (or items) are seen as manifestations of underlying latent constructs. Each construct of the model was measured by multiple reflective indicators that reflect individual experiences. In a reflective model, each indicator is assumed to be correlated with the underlying latent construct. The observed variables (indicators) are expected to vary together because of their shared connection with the construct they represent. Therefore, changes in the indicators (e.g., the ability to recognize and monitor physical or psychological changes) will reflect changes in the underlying constructs.

## 3. Study Design

### 3.1. Study Design and Study Setting

This study is a cross‐sectional survey that the reporting of results strictly adheres to the Strengthening the Reporting of Observational Studies in Epidemiology (StroBE) statement in terms of research design, data collection, data analysis, and reporting processes [39]. The reporting and writing also adhered to COnsensus‐based Standards for the selection of health status Measurement INstruments (COSMIN) criteria [40]. Convenient sampling was utilized for this study. We conducted this study in three hospitals in China. These sites included two tertiary general teaching hospitals in Jiangsu province and one in Liaoning Province, serving a diverse demographic of patients from both urban and surrounding rural areas. Data collection occurred from August to October 2024. The sample size for exploratory factor analysis (EFA) and confirmatory factor analysis (CFA) was determined based on established guidelines. To ensure rigorous cross‐validation, the total sample was divided into two independent datasets based on a temporal split. The first 161 participants recruited (Phase 1) were allocated to the EFA to explore the factor structure, while the subsequent 264 participants (Phase 2) were reserved for the CFA to validate the model. For EFA, a sample of 161 participants was used, which is within the recommended range of 5–10 participants per item for a 19‐item scale [41, 42]. This ensured stable factor extraction. For CFA, 264 participants were used, exceeding the typical minimum of 200, which provided sufficient power to confirm the factor structure and validate the model fit [41, 42]. A preprint of this paper has previously been published by Zheng et al. [43] in research square.

### 3.2. Eligibility Criteria

Inclusion criteria: (1) Age: Participants must be 18 or older to ensure legal consent and stable chronic conditions. (2) Dialysis frequency: Participants must have undergone regular hemodialysis for at least 3 months, with a minimum of one session per week, ensuring stable dialysis protocols. (3) Consent: Participants must provide informed consent and voluntarily agree to participate. Exclusion criteria: (1) Pending transplant or alternative dialysis: Individuals scheduled for a kidney transplant or planning peritoneal dialysis within a month are excluded to avoid confounding results. (2) Severe comorbid conditions: Individuals with severe conditions like active malignancies or psychiatric disorders are excluded, as these could distort HSB‐HD symptoms. (3) Communication barriers: Individuals unable to give informed consent or complete surveys because of cognitive impairments, severe hearing loss, or language barriers are excluded.

### 3.3. Patient Recruitment and Data Collection

Patient recruitment was managed by data coordinators (nurses or interns) at each subcenter, who underwent standardized training. Coordinators explained the survey details during one‐on‐one consultations, and participants who consented were provided either a paper questionnaire or a QR code linking to an online survey via Questionnaire Star. Coordinators were available for questions without influencing responses, and for patients without smartphones, paper versions were used. Data were uploaded to the Questionnaire Star platform for centralized management. This study was approved by the Medical Science Research Ethics Committee of the First Affiliated Hospital of China Medical University, with the ethical approval number 2024‐633.

### 3.4. Instruments

#### 3.4.1. General Demographic Data and Covariates Related to Scale Development

Demographic data and covariates were selected through literature review and expert panel, including gender, age, education, minority status, marital status, residence, solitude, socioeconomic status, income, smoking history, CKD history, dialysis center travel time, insurance type, work status, dialysis start time, and weekly dialysis frequency.

#### 3.4.2. Draft Version of HSB‐HD

The HSB‐HD was developed specifically for this study to assess the symptom HSB of patients undergoing hemodialysis. This scale is designed as a patient‐reported outcome measure (PROM) intended for self‐administration by the patient. The draft version of the HSB‐HD scale was designed to measure four key dimensions: Symptom detection, symptom interpretation, decision‐making for help‐seeking, and timely disclosure and taking action. The scale was developed following an extensive literature review, expert consultations, and patient cognitive interviews. The initial draft contained 19 items.

#### 3.4.3. EQ‐5D‐5L

The EQ‐5D‐5L is a standardized instrument for measuring HRQOL [44]. In this study, only the anxiety/depression dimension was used to assess the psychological aspects of health because of the theoretical implication. This dimension includes five levels of severity: No problems, slight problems, moderate problems, severe problems, and extreme problems.

#### 3.4.4. Short Form of Family Health Scale

The Family Health Scale is a 10‐item tool designed to assess the overall health and well‐being of family members, as well as their roles and support in managing chronic illness [45]. This scale includes items measuring family functioning, communication, and the ability of the family to cope with chronic illness challenges. While the Family Health Scale was considered for use in this study, it was not directly included in the analysis but may offer valuable contextual information regarding the patient’s support system. It provides an insight into how family health and functioning influence the patient’s decision‐making regarding seeking help for symptoms, especially in terms of the patient’s willingness to disclose symptoms or seek professional help when necessary.

#### 3.4.5. Chronic Illness Resource Survey (CIRS)

The CIRS is a tool designed to assess the resources available to individuals living with chronic illness, focusing on both tangible resources, such as health care access, and intangible resources, such as emotional and psychological support [46]. For this study, only the support from health care dimension of the CIRS was utilized because of the theoretical implication. This dimension measures the level of support patients receive from health care professionals, including the quality of communication, access to medical advice, and emotional support provided during treatment.

### 3.5. Item Generation and Revision

**FIGURE 2:**
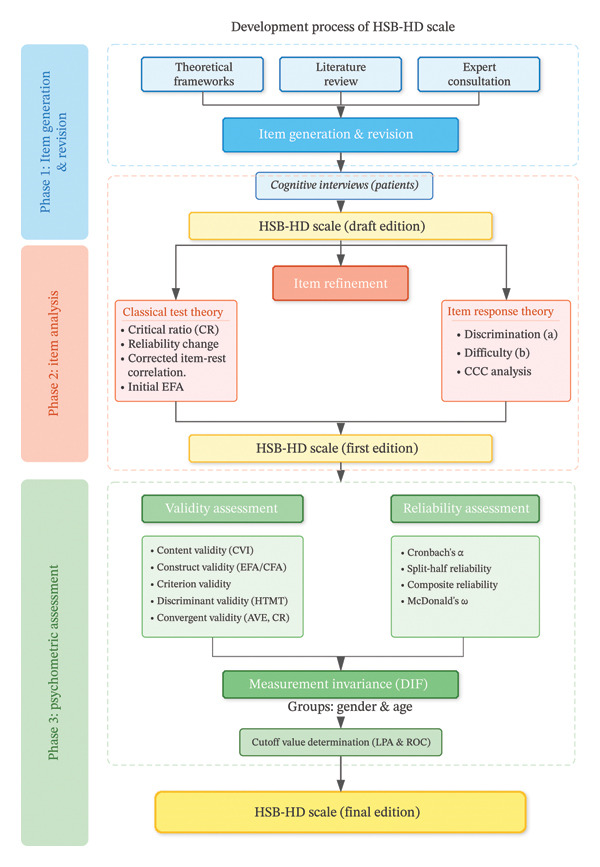
The whole development process of HSB.

To generate meaningful items, we first conducted a literature review by systematically searching databases and extracting relevant content from the literature. This was combined with the theoretical framework of the current study to form an initial set of items. To ensure both content validity and surface validity of the scale, we used a combination of expert review (expert panel meeting) and patient feedback (cognitive interviews) to supplement, refine, and optimize the item content. The whole development could be found in Figure 2.

Initially, we convened an expert consultation meeting to invite specialists from relevant fields to participate in the design and review of the items. Experts from the fields of epidemiology and statistics (*n* = 1), nursing (*n* = 2), nephrology (*n* = 2), behavioral medicine (*n* = 1), and psychology (*n* = 1) were invited to participate. During the meeting, we provided a detailed explanation of the background and theoretical foundations of the scale development and asked experts to revise or propose items based on the theoretical dimensions. We then synthesized, summarized, and combined the feedback from multiple experts to form the first draft of the scale.

Following this, we used cognitive interviews to further revise and complement the items [47]. A purposive sampling method was employed, considering differences in patients’ age, economic status, and educational level. Before the interviews, patients were informed of the research purpose and asked to complete the questionnaire. Semi‐structured interviews were then conducted based on an interview guide. The interview questions were as follows: (1) How do you understand this item? (2) What were you thinking when answering this question? (3) Why did you choose this option? (4) Was it easy for you to choose this option? (5) Is there anything that needs to be improved in this item? (6) Are there any other issues related to symptom help‐seeking that the questionnaire has not addressed but should include? (7) Do you have any suggestions for modifying the entire questionnaire? After the interviews, the researchers used content analysis to summarize the issues raised by the patients, and based on the findings, the questionnaire was revised. The modified content was then presented again to the patients for confirmation to ensure that the changes reflected their original intentions.

### 3.6. Item Analysis

In the item analysis process, a variety of statistical methods were employed to conduct item reduction. Initially, classical test theory (CTT) methods were applied to assess the reliability and discrimination of the items. The critical ratio (CR) method was used to evaluate item discrimination by comparing the performance of the top and bottom 27% of participants. To calculate the CR, each item’s mean score for the high and low groups is calculated, and the difference between these means is divided by the standard error of the difference. The CR value can be interpreted as follows: items with a CR value above 3.0 could indicate the item effectively differentiates between high and low performers [48].

Next, the reliability change if an item is dropped was assessed. For each item, Cronbach’s alpha (*α*) and McDonald’s *ω* was computed for the overall scale and for the scale with the item removed. If dropping an item significantly increases the Cronbach’s alpha, it suggests that the item is not contributing positively to the internal consistency of the scale and need to be reconsidered for exclusion.

The corrected item‐rest correlation was also computed, which measures the correlation of each item with the total score (excluding that item). Higher item‐rest correlations indicate better item performance, meaning the item is closely related to the overall construct being measured. Items with low item‐rest correlations (below 0.40) may not be effectively capturing the underlying trait and may be candidates for removal [49].

Furthermore, an initial EFA was conducted to explore the factor structure of the scale. In this analysis, factor loadings were calculated for each item, reflecting the strength of the relationship between the item and the underlying factor. Items with low factor loadings (below 0.40) are considered weak indicators of the factor and should be excluded from the final scale [50].

For item response theory (IRT), we assessed the ratio of the first to the second eigenvalue from the EFA. A ratio greater than 3.00 indicates that the data meet the unidimensionality assumption [51]. Consequently, the graded response model (GRM) was used with marginal maximum likelihood estimation (MLE) to calculate the *α* and *b* parameters for each item, and item information was calculated. The *α* parameter represents the discrimination power of the item, reflecting its ability to distinguish between different levels of the trait. An *α* value between 0.3 and 3.0 was considered acceptable; values that are too low lack sufficient discrimination, while values that are too high limit the precision of measurement [51]. The *b* parameter represents the difficulty level, with higher *b* values indicating more difficult items. In this study, *b* values were set within the range of −3 to 3. Items outside this range were excluded, and the difficulty levels of item categories should follow a monotonic increasing trend [51].

To further assess item performance, category characteristic curves (CCC) were examined [52]. For a Likert five‐point scale, the curves for the extreme categories (1 and 5) should show monotonic characteristics, meaning the probability of selecting these categories increases or decreases smoothly as the latent trait increases. However, for the middle categories (2, 3, and 4), the curves tend to exhibit a bimodal shape, resembling a peak. This behavior indicates that respondents with moderate levels of the latent trait may be more likely to select one of these middle categories, whereas respondents with higher or lower levels of the trait are more likely to select the extreme categories (1 or 5). Items that show irregular or nonmonotonic curves for the extreme categories or have flat peaks for the middle categories should be excluded, as they fail to provide meaningful information across the full range of the trait [52].

### 3.7. Validity Assessment

The validity of the scale was assessed using four key methods: content validity, criterion validity, discriminant validity, and convergent validity. Each of these methods was used to ensure that the scale accurately measures the intended constructs and differentiates between related and unrelated constructs.

#### 3.7.1. Content Validity

Content validity was assessed by examining the relevance and representativeness of the items within the scale relative to the construct being measured. This was done using the item‐content validity index (I‐CVI), which was calculated by having a panel of experts we invited to evaluate each item on a four‐point scale (1 = not relevant, 4 = highly relevant). Items with an I‐CVI score of 0.78 or higher were considered to have adequate content validity [53]. The overall scale’s content validity was then assessed by computing the scale‐content validity index (S‐CVI), which is the average of the I‐CVIs for all items in the scale.

#### 3.7.2. Construct Validity

Construct validity was assessed using both EFA and confirmatory factor analysis (CFA) to examine the factor structure and validate the underlying construct(s) measured by the scale.

Before conducting the EFA, assumptions were checked to ensure the data were suitable for factor analysis. The Kaiser–Meyer‐Olkin (KMO) measure was used to assess sampling adequacy. A KMO value greater than 0.60 was considered acceptable for conducting factor analysis [50]. In addition, Bartlett’s test of sphericity was performed to test whether the correlation matrix is significantly different from an identity matrix. A significant result (*p* < 0.05) in Bartlett’s test indicates that the variables are sufficiently correlated to proceed with factor analysis [50]. Principal axis factoring (PAF) with Promax rotation was used for EFA. This method was selected because it is robust for identifying latent variables, particularly when the data are not perfectly normally distributed, as is typical with Likert scale data. To determine the number of factors to retain, parallel analysis was applied, which compares the eigenvalues from the data to those obtained from random data [54]. Factors with eigenvalues greater than those derived from the random data were retained. This method is considered more accurate than the traditional eigenvalue‐greater‐than‐1 rule and helps ensure an appropriate number of factors are selected [54].

The factor loadings were examined to assess the strength of each item’s association with its respective factor. Items with loadings less than 0.40 were considered weak indicators of their respective factor and were considered for removal [50]. Cross‐loadings were not allowed for each item [50].

After EFA, confirmatory factor analysis (CFA) was performed to validate the factor structure. CFA was carried out using MLE to test how well the proposed model fits the observed data. The following fit indices were used to assess model fit [55, 56]: (1) Chi‐square (*χ* ^2^): A significant chi‐square indicates a poor model fit, though it is sensitive to sample size. (2) Comparative fit index (CFI): A value above 0.90 suggests a good fit. (3) Root mean square error of approximation (RMSEA): Values below 0.08 suggest an acceptable fit, with values below 0.05 indicating a good fit. (4) Standardized root mean square residual (SRMR): Values below 0.08 indicate a good fit.

#### 3.7.3. Criterion Validity

Criterion validity was assessed using conceptually relevant variables from the HSB model, as no existing scale for hemodialysis symptoms was available. Antecedent variables, such as family health total score and support from health care total score, were selected based on their theoretical relevance to HSB [36]. These variables reflect personal and external resources that may affect the individual’s decision to seek help in managing symptoms associated with hemodialysis. Consequent variables, including EQ‐5D‐5L Pain, EQ‐5D‐5L Anxiety, and EQ‐5D‐VAS, were used to evaluate the impact of seeking help on health outcomes [36]. Correlations between these antecedents and consequences were analyzed to validate the scale’s ability to measure HSB.

#### 3.7.4. Discriminant Validity

Discriminant validity was assessed to ensure that the scale measures distinct constructs and does not show excessive correlations with unrelated constructs [57]. In this study, Heterotrait–Monotrait Ratio (HTMT) was used to evaluate discriminant validity. The HTMT is a more advanced method that compares the average of the correlations between items from different constructs (heterotrait) with the average of the correlations between items from the same construct (monotrait). To assess discriminant validity, the HTMT ratio should be less than 0.9, which is generally considered an indication that the constructs are distinct. If the HTMT value exceeds 0.9, it suggests that there may be a potential overlap in the theory aspect [57].

#### 3.7.5. Convergent Validity [average variance extracted (AVE) and CR]

Convergent validity was assessed by examining the degree to which items on the scale measure the same construct. This was evaluated using two indices: AVE and CR. AVE represents the average amount of variance in the items that is explained by the latent construct. An AVE value above 0.50 indicates that the construct explains more than half of the variance in the items, supporting convergent validity [58].

### 3.8. Reliability Assessment

Reliability was assessed using three common methods: Cronbach’s alpha (*α*), split‐half reliability coefficient, and composite reliability. Cronbach’s alpha was calculated to assess the internal consistency of the scale [59]. This coefficient measures the degree to which items on the scale are interrelated, with higher values indicating better internal consistency. A value of *α* ≥ 0.70 is generally considered acceptable for establishing reliability [59]. For this study, Cronbach’s alpha was computed for the entire scale, as well as for individual subscales, to evaluate the reliability of each component. Split‐half reliability was assessed by dividing the items on the scale into two equal parts, either randomly or by odd/even item grouping. The correlation between the two halves of the scale was computed to determine the reliability of the scale. The Spearman–Brown prophecy formula was applied to adjust the correlation [60]. A coefficient of 0.70 or higher was considered acceptable for split‐half reliability. Composite reliability assesses the internal consistency of the construct’s items. A CR value of 0.70 or higher indicates that the items are reliably measuring the same underlying construct.

### 3.9. Measurement Invariance Assessment Using IRT

Measurement invariance was tested across different gender and age groups (cutoff value of age is 60) using IRT to examine whether the scale functions similarly across these groups [52]. The analysis was conducted using the adjacent model, which is appropriate for the ordinal nature of the data. Both options for differential item functioning (DIF) were selected to assess whether the items function differently for each group across both the adjacent response categories and overall.

The Z‐score matching criterion was used to standardize the comparison across groups, allowing for a direct comparison of item performance across different demographic groups. The Benjamini–Hochberg (BH) method was chosen for multiple comparison correction to control the false discovery rate in the DIF analysis. An item purification process was performed to refine the items by identifying and removing those that exhibit significant DIF, ensuring that the final set of items is invariant across the different groups [52]. For judging DIF, adjusted *p* values were used. Items with an adjusted *p* value below 0.05 were considered to exhibit significant DIF, indicating that the item functions differently across the demographic groups (gender or age) [52].

### 3.10. Determination of the Cutoff Value for the Scale

Latent profile analysis (LPA) and receiver operating characteristic (ROC) analysis were employed to establish the cutoff value within the dataset. LPA is a statistical technique used to categorize individuals into distinct groups based on their response patterns to observed variables [61]. The analysis was conducted using the tidyLPA package in R. The optimal number of latent classes was identified by comparing models with different numbers of classes (2, 3, 4, etc.) using three primary model fit criteria: the Akaike information criterion (AIC), Bayesian information criterion (BIC), and entropy. Both AIC and BIC are used to assess the fit of the model while accounting for its complexity [61]. These indices favor simpler models, with lower values indicating better fit. When comparing models with varying numbers of latent classes, the model with the lowest AIC and BIC values is generally considered the most optimal. Entropy, on the other hand, gauges the clarity of classification; higher entropy values indicate that individuals are more distinctly assigned to specific latent classes. A value approaching one indicates well‐defined groups, while a value near zero suggests weaker group distinctions [61].

In this study, the optimal cutoff value for identifying latent classes related to the digital divide was determined through ROC analysis [62, 63]. This involved comparing pairs of categories at a time by merging adjacent latent classes to create binary outcomes, which facilitated the calculation of cutoff values distinguishing different degrees of the digital divide [62, 63]. The Youden’s index was applied to identify the optimal cutoff, as it maximizes the combined sensitivity and specificity of the test. Sensitivity corresponds to the true positive rate, while specificity refers to the true negative rate. The Youden’s index is calculated as the sum of sensitivity and specificity minus one. The cutoff value associated with the highest Youden’s index was selected as the most effective threshold for classifying individuals based on their degree of digital divide [62, 63].

## 4. Results

### 4.1. Demographic Characteristics

A total of 425 participants were surveyed, with the first phase of 161 being used for item analysis and EFA, and the subsequent 264 being used for CFA. As shown in Table 2, of the total sample, 54.80% were male (*n* = 233) and 45.20% were female (*n* = 192). The largest age group was 51–60 years (27.80%, *n* = 118), followed by > 60 years (26.10%, *n* = 111). Most participants had a junior college education or below (81.60%, *n* = 347). The majority were married (78.10%, *n* = 332), and 54.80% lived in urban areas (*n* = 233). Regarding income, 46.40% earned less than 3000 (*n* = 197), and 61.60% had never smoked (*n* = 263). For CKD causes, glomerulonephritis (30.10%, *n* = 128) was the most common, and most participants (80.90%, *n* = 344) received dialysis three times per week. Most participants (44.50%, *n* = 189) had been on dialysis for 1–5 years, and 53.20% had medical insurance for residents (*n* = 226). In terms of socioeconomic status, the majority rated themselves at level 4 (24.00%, *n* = 102), followed by level 3 (19.30%, *n* = 80).

**TABLE 2:** Characteristic of demographic information.

Demographic information	Level	Count	Proportion (%)
Gender	Male	233	54.80
	Female	192	45.20

Age	18–25	20	4.70
	26–30	9	2.10
	31–35	37	8.70
	36–40	45	10.60
	41–50	85	20.00
	51–60	118	27.80
	> 60	111	26.10

Education level	Junior college and below	347	81.60
	Undergraduate	69	16.20
	Postgraduate	9	2.10

Minority	Yes	53	12.50
	No	372	87.50

Marital status	Married	332	78.10
	Unmarried	54	12.70
	Divorced	23	5.40
	Widowed	16	3.80

Residence	Urban	233	54.80
	Urban‐rural fringe area	111	26.10
	Rural	81	19.10

Solitude state	Yes	78	18.40
	No	347	81.60

Socioeconomic status (self‐rated)	0	17	4.00
	1	40	9.40
	2	56	13.20
	3	82	19.30
	4	102	24.00
	5	64	15.10
	6	36	8.50
	7	28	6.60

Average income per person	< 3000	197	46.40
	3000–3999	87	20.50
	4000–4999	64	15.10
	> 5000	77	18.10

History of smoking	Never	263	61.90
	Quit smoking now	99	23.30
	Smoking now	63	14.80

Protopathy of the chronic kidney disease	Glomerulonephritis	128	30.10
	Hypertension	117	27.50
	Diabetes	91	21.40
	Unsure	89	20.90

Time travel to dialysis center	< 30 min	164	38.60
	30–60 min	143	33.60
	> 60 min	118	27.80

Medical insurance type	Medical insurance for residents	226	53.20
	Medical insurance for employees	169	39.80
	Else	30	7.10

Working status	Working	86	20.20
	Off duty	339	79.80

Time starting dialysis	< 1 year	110	23.6
	1–5 years	189	44.50
	6–10 years	77	18.10
	> 10 years	59	13.90

Dialysis times per week	1 time per week	28	6.60
	2 times per week	34	8.00
	3 times per week	344	80.90
	5 times per 2 weeks	19	4.50

### 4.2. Results of Item Generation and Revision

The items in this study were derived from three sources: Literature review, expert meetings, and cognitive interviews with patients. Among them, nine items (47.37%) were generated from the literature review, seven items (36.84%) from expert meetings, and three items (15.79%) from cognitive interviews with patients. A total of 10 patients and 6 experts were included in the study, and the demographic information of the patients and experts can be found in the Supporting File 1. In total, four items (21.05%) were revised by experts, and five items (26.32%) were modified based on cognitive interviews with patients. The process and detailed information of each item’s generation and revision are provided in the Supporting File 1.

### 4.3. Results of Item Analysis

Item analysis was conducted to evaluate the performance of each item in the scale and to reduce items based on their contribution to the overall scale (see Table 3). The analysis was based on both CTT and IRT parameters. All CR values for each item exceeded 3.0. For internal consistency, Cronbach’s *α* if item dropped ranged from 0.953 to 0.956, and McDonald’s *ω* if item dropped ranged from 0.954 to 0.957. No internal consistency parameters were detected improved after any item was removed. Factor loadings were assessed, with most items showing adequate loadings above 0.588, indicating that the items contributed well to the construct being measured. Item 5, however, had the lowest factor loading (0.322) and was consequently deleted from the scale because of its insufficient contribution. Corrected item‐total correlations were also examined, ranging from 0.588 to 0.794, indicating that most items were strongly correlated with the total scale score.

**TABLE 3:** Item analysis results.

Item number	CTT					IRT					Retainment of item
	Differential functioning	Internal consistency‐reliability change if item dropped		Discrimination	Factor loading	Discrimination	Difficulty thresholds				
	CR	Cronbach’s *α*	McDonald’s *ω*	Corrected item‐total correlation			2 VS 1	3 VS 2	4 VS 3	5 VS 4	
1	−12.5	0.956	0.957	0.588	0.886	1.870	−1.938	−1.354	−0.251	1.499	✔
2	−12.8	0.956	0.957	0.598	0.931	1.870	−1.919	−1.390	−0.277	1.730	✔
3	−14.5	0.955↓	0.956↓	0.643	0.643	1.870	−1.835	−1.142	−0.049	1.982	✔
4	−13.2	0.954↓	0.956↓	0.678	0.658	1.870	−1.906	−1.682	−0.251	1.833	✔
5	−7.96	0.955↓	0.956↓	0.658	**0.322**	1.870	−2.353	−1.631	−0.566	1.670	×
6	−16.6	0.954↓	0.955↓	0.734	0.696	1.870	−2.886	−1.738	−0.226	1.652	✔
7	−16.8^a^	0.954↓	0.955↓	0.725	0.846	1.870	−2.809	−1.474	−0.267	1.862	✔
8	−15.9	0.953↓	0.955↓	0.747	0.786	1.870	−2.462	−1.674	−0.387	1.721	✔
9	−16.4^a^	0.953↓	0.954↓	0.764	0.529	1.870	−1.977	−1.542	−0.293	1.733	✔
10	−16.8	0.953↓	0.954↓	0.772	0.589	1.870	−1.944	−2.024	−0.544	1.464	×
11	−17.1	0.953↓	0.954↓	0.778	0.79	1.870	−2.573	−1.713	−0.408	1.700	✔
12	−16.9	0.953↓	0.954↓	0.756	0.734	1.870	−2.627	−1.588	−0.289	1.637	✔
13	−17.7	0.953↓	0.954↓	0.77	0.509	1.870	−2.450	−1.704	−0.380	1.605	✔
14	−16.8	0.953↓	0.954↓	0.765	0.711	1.870	−2.393	−1.809	−0.535	1.270	✔
15	−17	0.953↓	0.954↓	0.762	0.794	1.870	−2.396	−1.793	−0.586	1.199	✔
16	−15.7	0.953↓	0.954↓	0.757	0.945	1.870	−2.295	−1.982	−0.712	1.072	✔
17	−14.9	0.954↓	0.955↓	0.715	0.928	1.870	−2.356	−1.855	−0.721	1.171	✔
18	−15.6	0.954↓	0.955↓	0.737	0.814	1.870	−2.498	−1.875	−0.498	1.332	✔
19	−14.1	0.954↓	0.956↓	0.68	0.561	1.870	−2.315	−1.752	−0.352	1.476	✔

*Note:* 0.322 means the factor loading of this item is too low to support this item belonging to this domain.

**FIGURE 3:**
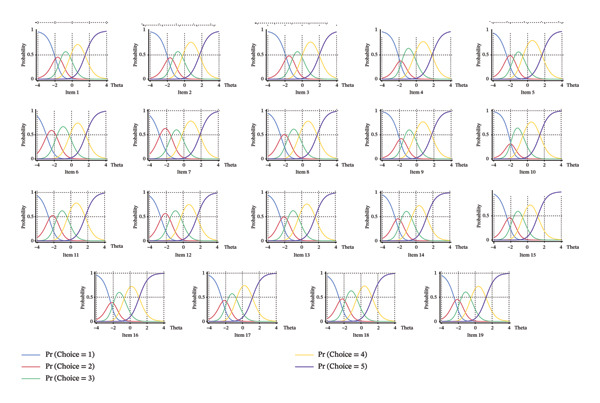
Category characteristic curves.

The analysis also included IRT parameters, which assessed the discrimination ability and difficulty levels of the items (see Table 3). The ratio of the first to the second eigenvalue from the EFA was 6.67, greater than 3.00, indicating that the data meet the unidimensionality assumption. The overall discrimination index for the entire scale was 1.870, indicating that, on average, the items were effective in distinguishing between participants with different levels of the construct. The high discrimination values indicate that these items are particularly effective at identifying “tipping points” in patient behavior, providing the sensitivity needed to accurately classify patients into specific behavioral profiles (e.g., distinguishing between “context‐dependent” and “proactive” help‐seekers). Difficulty thresholds ranged from −2.886 to 1.499, with most items falling within a suitable difficulty range, ensuring that they were appropriate for distinguishing between participants with different levels of the construct. This broad range of difficulties suggests that the scale is robust across the clinical spectrum, capable of capturing “easy” behaviors performed by most patients (such as basic symptom detection) as well as “difficult” actions (such as timely disclosure) that are only undertaken by highly proactive individuals. Item 10 violated the basic assumptions of IRT. Specifically, its response patterns did not conform to the expected monotonicity of a Likert scale, indicating potential issues with how participants interpreted the item. Because of this violation, item 10 was also removed from the final scale. This lack of monotonicity suggests that Item 10 was likely ambiguous or interpreted inconsistently by patients, causing those with higher help‐seeking tendencies to select lower response categories unpredictably. Removing such items is crucial to ensure that the total score accurately and linearly reflects the patient’s actual level of HSB. Besides, most items had thresholds within an acceptable range, ensuring that the scale was effective in capturing varying levels of the construct (see Figure 3).

### 4.4. Results of Validity Assessment

#### 4.4.1. Content Validity

The content validity of the scale was assessed, and the results indicated strong content relevance. Out of the 17 items, 13 items achieved an I‐CVI score of 1, indicating high relevance to the construct. Four items (items 4, 9, 12, and 14) received an I‐CVI score of 0.86, also demonstrating satisfactory relevance. The overall S‐CVI is 0.96, which confirmed the scale’s robust content validity. The score of each item appraised by the expert are provided in the Supporting File 2.

#### 4.4.2. Construct Validity

**FIGURE 4:**
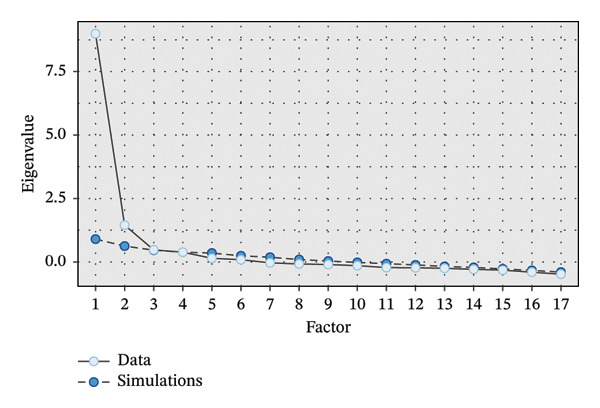
Parallel analysis results.

EFA revealed a four‐factor structure that accounted for 72.9% of the total variance. Factor loadings ranged from 0.416 to 0.975. The parallel analysis results demonstrated a clear four‐factor structure that aligns with the intended constructs (Figure 4). CFA was conducted to validate the factor structure derived from the EFA. The fit indices from CFA showed a good model fit (see Table 4). These values suggest that the proposed model fits the observed data well. Consequently, we did not do any modification to the model.

**TABLE 4:** Model fit value of reference and four‐factor model.

Model fit names	Reference value	Vale of the 4‐factor model	Interpretation with rating
RMSEA	< 0.08	0.049 95% CI (0.036, 0.061)	Good
Standardized root mean square residual (SRMR)	< 0.08 acceptable < 0.05 good	0.034	Good
Comparative fit index (CFI)	> 0.90 acceptable > 0.95 good	0.967	Good
Tucker Lewis index (TLI)	> 0.90 acceptable > 0.95 good	0.960	Good

#### 4.4.3. Criterion Validity

**FIGURE 5:**
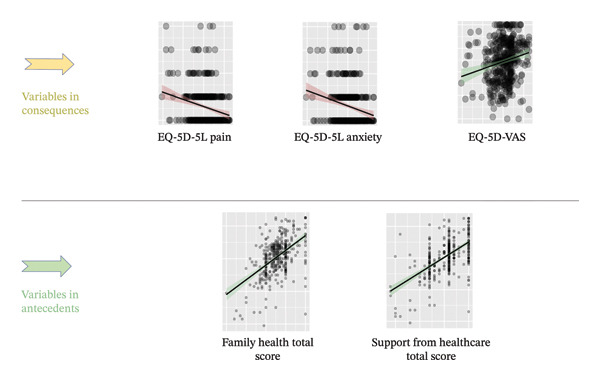
Results of criterion validity.

The HSB scale demonstrated significant correlations with related variables (Figure 5). It showed a negative correlation with EQ‐5D pain (*r* = −0.210, *p* < 0.001) and EQ‐5D anxiety (*r* = −0.205, *p* < 0.001), indicating that higher levels of HSB were associated with lower reported pain and anxiety. In addition, a positive correlation was found with EQ‐5D VAS (*r* = 0.192, *p* < 0.001), suggesting that individuals engaging in higher levels of HSB had better overall health status.

Significant positive correlations were also found with antecedent variables, including family health (*r* = 0.520, *p* < 0.001) and support from health care (*r* = 0.569, *p* < 0.001), which are known to influence HSB.

#### 4.4.4. Discriminant Validity and Convergent Validity

Discriminant validity was established, as most of the HTMT values for all factors were below the threshold of 0.90, confirming that the constructs were distinct. However, the HTMT between Factors 2 and 3 has exceeded 0.9, indicating that these two constructs may have some overlap variance in the aspect of theory or statistic. Convergent validity was supported by the AVE. The AVE for all four factors exceeded the 0.50 threshold, indicating that the items explained more than half of the variance in their respective constructs.

### 4.5. Results of Reliability Assessment

Cronbach’s alpha for the entire scale was 0.953, indicating excellent internal consistency. The subscales also demonstrated strong reliability, with the following values: symptom detection (*α* = 0.906), symptom interpretation (*α* = 0.875), decision‐making for help‐seeking (*α* = 0.859), and timely disclosure and taking action (*α* = 0.938). All these values exceeded the acceptable threshold of 0.70, supporting the internal consistency of the scale and its components. For split‐half reliability, the scale was divided into two equal parts. The reliability for the first half was 0.916, while the second half showed a reliability of 0.938. The Spearman–Brown prophecy formula adjusted the correlation, yielding a coefficient of 0.856 for both equal and unequal lengths, further confirming the reliability of the scale. The CR values ranged from 0.801 to 0.883, demonstrating excellent internal consistency and reliability for the scale.

### 4.6. Results of Measurement Invariance of the Scale

The measurement invariance of the scale was tested across gender and age groups using IRT, with significant DIF assessed based on the adjusted *p* values (Adj.*p*) (see Table 5). For gender, significant DIF was observed in two items: item 2 (Adj.*p* = 0.011) and item 17 (Adj.*p* = 0.043), indicating that these items functioned differently across gender groups. The other items, including items 1, 3, 4, 5, 6, 7, 8, 9, 10, 11, 12, 13, 14, 15, and 16, did not show significant DIF, as their adjusted *p* values were above 0.05. For age groups (cutoff at 60 years), no items exhibited significant DIF. All items had adjusted *p* values greater than 0.05, indicating no differential functioning across the age groups. The final edition of the scale could be found in Supporting File 3.

**TABLE 5:** Differential item functioning of the scale.

	Gender			Age		
	Statistic	*p*	Adj.*p*	Statistic	*p*	Adj.*p*
1	4.784	0.091	0.222	6.9279	0.031	0.532
2	14.616	< 0.001	0.011	3.7334	0.155	0.798
3	0.34	0.844	0.893	1.4651	0.481	0.802
4	5.313	0.07	0.222	0.3086	0.857	0.969
5	0.226	0.893	0.893	3.0837	0.214	0.798
6	0.294	0.863	0.893	0.7674	0.681	0.891
7	1.659	0.436	0.707	0.1512	0.927	0.969
8	2.59	0.274	0.582	2.2486	0.325	0.798
9	0.263	0.877	0.893	2.6199	0.27	0.798
10	2.278	0.32	0.605	1.0451	0.593	0.84
11	8.698	0.013	0.073	2.0896	0.352	0.798
12	1.231	0.54	0.707	1.959	0.375	0.798
13	4.883	0.087	0.222	1.3855	0.5	0.802
14	1.302	0.522	0.707	0.3858	0.825	0.969
15	1.432	0.489	0.707	0.062	0.969	0.969
16	5.112	0.078	0.222	2.9055	0.234	0.798
17	10.555	0.005	0.043	1.3125	0.519	0.802

*Note:* Adj.*p* = the adjusted *p* values by likelihood ratio test using multiple comparison.

### 4.7. Cutoff Value of the Scale

**FIGURE 6:**
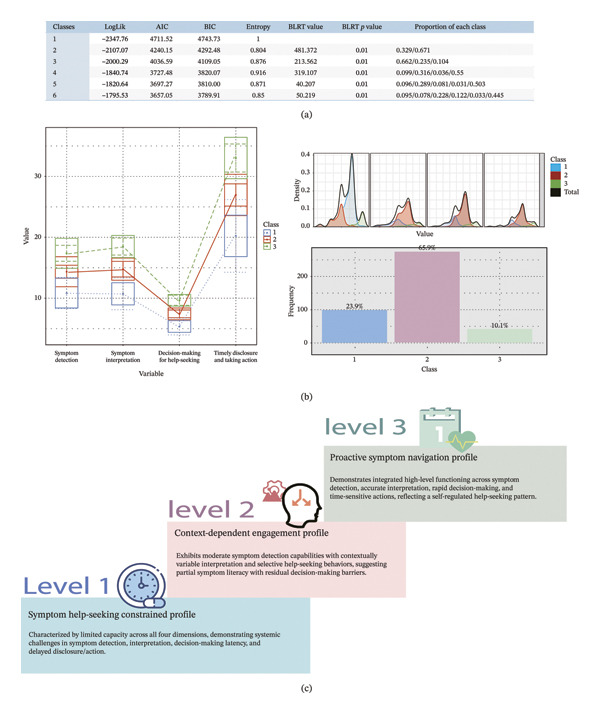
Cut off value selection.

From Figure 6(a), it can be observed that as the number of profiles increases, the values of AIC and BIC decrease gradually. The entropy values are higher for the three‐class and four‐class models, with the highest entropy occurring at four classes. The likelihood ratio test indicates that models with 2–6 profiles are all acceptable. However, we observed that for the four‐class model and those with a higher number of profiles, there were categories with proportions smaller than 5%, suggesting that these classifications are unreasonable. In conclusion, based on both statistical analysis and interpretability of the results, we selected the three‐class model as the final categorization. Figure 6(b) presents three panels from the LPA across three classes. The boxplots (top‐left) show that Class 1 (blue) has lower values, Class 2 (red) has intermediate values, and Class 3 (green) has higher values. The density and frequency bar plots (top‐right and bottom) reveal that Class 2 (red) is the most common, with the highest density and comprising 65.9% of the sample, while Class 1 (blue) makes up 23.9% and Class 3 (green) 10.1%. The cutoff values for the three latent symptom HSB categories, 56 and 72, were determined using ROC analysis and Youden’s index to maximize sensitivity and specificity between adjacent classes. The three latent groups of symptom HSB scale were named: HSB constrained profile, context‐dependent engagement profile, and proactive symptom navigation profile (Figure 6(c)).

## 5. Discussion

### 5.1. Innovativeness and Scientific Foundation of the Scale

The HSB‐HD developed in this study was proposed against the backdrop of the growing global burden of kidney disease. As CKD has become one of the leading causes of death worldwide, symptom management for this patient group is particularly critical. Enhancing and optimizing patients’ HSBs is a key component of symptom management. The development of the HSB‐HD scale responds to the call from the kidney disease: improving global outcomes (KDIGO) consensus for symptom assessment and management, aiming to provide an effective tool for assessing the HSB intentions of hemodialysis patients. This, in turn, will improve the weaknesses in their HSBs, reduce symptom burden, and enhance HRQOL.

### 5.2. Scale Development and Validation Process

The development and validation process of the HSB‐HD scale, as detailed in the methodology section, reflects rigorous scientific and systematic approaches. This study is based on Maryam Momeni’s conceptual model of HSB, and the HSB‐HD scale considers key attributes of the symptom detection, interpretation, disclosure, and help‐seeking decision‐making processes. Through factor analysis, we found that the original dimensions of “taking action to seek help” and “help‐seeking decision” were highly correlated. Based on the explanation of the CSM of self‐regulation, we combined these two dimensions. This adjustment enriches and refines the fundamental concepts of Momeni’s model of HSB.

The generation of scale items was based on an extensive literature review and preliminary theoretical research, ensuring the relevance and comprehensiveness of the content. Through expert consultations and cognitive interviews with patients, we initially developed the scale items, which were revised and optimized based on feedback. The expert team consisted of specialists in the fields of epidemiology, nursing, nephrology, behavioral medicine, and psychology. The expert meetings ensured the scientific accuracy and comprehensiveness of the scale’s content. In addition, the cognitive interviews with patients enhanced the surface validity of the scale, ensuring its adaptability and comprehensibility for the target population. Furthermore, both classical measurement theory and the more stringent IRM were used for item reduction, ensuring that the retained items demonstrated stable psychometric properties.

This study employed a series of validation methods to test the reliability and validity of the scale, including content validity, criterion‐related validity, discriminant validity, and convergent validity. Content validity was determined through expert review, with each item being rigorously assessed to ensure its relevance to hemodialysis HSB. Exploratory and confirmatory factor analyses further tested the construct validity of the scale, while IRT was used to evaluate the discriminative ability and difficulty level of each item. For criterion‐related validity, the scale’s correlations with EQ‐5D‐5L pain, anxiety, and EQ‐5D‐VAS scales were tested, verifying its sensitivity to the impact of hemodialysis on patients’ quality of life. In addition, measurement invariance was examined across different gender and age groups to ensure consistent measurement performance across diverse populations.

### 5.3. Practical Significance in Clinical Application

The clinical practicality of the HSB‐HD scale lies in its ability to assist medical teams in identifying and screening hemodialysis patients who may encounter difficulties at certain stages of the help‐seeking process. Through systematic evaluation of patients’ HSB, clinicians can intervene earlier, providing personalized support and interventions to alleviate patients’ symptom burden. Furthermore, the assessment based on this scale may also enhance communication between patients and health care providers, enabling patients to more actively participate in their own treatment process.

Symptom detection refers to the process by which patients become aware of changes or symptoms in their bodies [36]. To enhance symptom detection, health care teams can use educational interventions to increase patients’ sensitivity to bodily signals. For example, regular health education sessions can be conducted to introduce common symptoms caused by hemodialysis and their physiological mechanisms, helping patients identify and monitor these symptoms [64]. In addition, providing tools for daily monitoring, such as symptom diaries or mobile applications, can help patients record and track changes in their symptoms, enabling timely identification of issues [65, 66].

Symptom interpretation refers to the process by which patients interpret these symptoms [36]. Improving symptom interpretation requires providing more information and resources to help patients accurately understand their symptoms. Health care providers can regularly assess whether patients’ interpretations of their symptoms are accurate and correct misunderstandings through one‐on‐one counseling. It is recommended to create personalized informational handbooks or educational videos that explain the possible causes of various symptoms and strategies for managing them, thereby enhancing patients’ self‐management capabilities [67].

Decision‐making for help‐seeking refers to the process by which patients decide whether to seek help based on their interpretation of symptoms [36]. To improve decision‐making for help‐seeking, health care teams can intervene with psychological support and decision‐making skills enhancement. For instance, psychological counseling can help patients address anxiety and fear related to the disease, which often influence their help‐seeking decisions [68]. In addition, decision aids can be introduced to assist patients in making more informed help‐seeking decisions after understanding the potential benefits and risks of various interventions [69].

Timely disclosure and taking action refers to the process by which patients promptly disclose their symptoms to obtain appropriate medical help and take actual steps to seek assistance [36]. To promote timely disclosure and action, health care teams should establish an open and supportive communication environment. Regularly check whether patients have any unreported symptoms and encourage them to share any concerns or discomforts. Furthermore, health care teams can design simplified help‐seeking processes, such as setting up dedicated hotlines, online consultation services, or emergency assistance channels, to ensure that patients can quickly access help when needed [70].

The identification of cutoff values (56 and 72) for the HSB‐HD scale through LPA and ROC analysis provides a clinically actionable framework for stratifying hemodialysis patients into three distinct behavioral profiles: Symptom help‐seeking constrained (23.9%), context‐dependent engagement (65.9%), and proactive symptom navigation (10.1%). The selection of a three‐class model balanced statistical rigor (evidenced by declining AIC/BIC values and maximized entropy) with clinical interpretability, avoiding over‐fragmentation into impractical subgroups. The cutoff derivation via Youden’s index prioritized optimizing both sensitivity (identifying true positives) and specificity (excluding false negatives), enabling clinicians to tailor interventions based on behavioral phenotypes. For instance, symptom help‐seeking constrained patients, characterized by limited symptom recognition and disclosure, may benefit from structured symptom‐monitoring tools (e.g., digital diaries) and health literacy programs to enhance self‐awareness [71, 72]. Conversely, context‐dependent engagement individuals, who seek help variably depending on external cues, might require family‐mediated support systems and routine clinician–patient communication protocols to reduce situational barriers [73]. Notably, the low prevalence of the proactive symptom navigation profile (10.1%) underscores a critical gap in autonomous symptom management, suggesting systemic barriers—such as fear of medical burden or inadequate access to resources—that warrant targeted psychosocial interventions.

To implement these interventions in a real‐world clinical setting without increasing the burden on nursing staff, we recommend integrating them into the existing dialysis workflow. For instance, the “digital diaries” suggested for the constrained profile can be completed by patients during the pre‐dialysis waiting period, effectively utilizing this idle time. The review of these diaries can then be synchronized with the nurse’s standard vascular access assessment or safety checks at the start of the session, ensuring that symptom data directly informs immediate clinical decisions (e.g., ultrafiltration adjustments). For the context‐dependent profile, the “Symptom Rounds” can be operationalized by designating a specific “communication window” during the first hour of treatment—when patients are typically most alert—explicitly inviting symptom disclosure as a standard component of the care protocol rather than an ad hoc request.

### 5.4. Limitations of the Study and Future Research Directions

In spite of the significant results achieved in this study, several limitations exist. The sample was limited to three hospitals in China, which may affect the generalizability of the findings. Future research should expand to more regions and cultural contexts to validate the cross‐cultural applicability of the scale. In addition, this study utilized a cross‐sectional design, which limits the ability to track long‐term trends in HSB. Therefore, longitudinal studies are recommended in future research to explore the dynamic relationship between HSB and clinical outcomes in patients. Furthermore, although the number of items in the scale developed in this study is moderate, considering the low health literacy of some dialysis populations and the need for repeated measurements in large cohorts, future research should develop a shortened version of the HSB‐HD scale or computer adaptive testing to further reduce the measurement burden for these populations. Finally, we acknowledge a potential for social desirability bias given that data collection was facilitated by data coordinators (nurses or interns). Although we managed this by providing standardized training to ensure coordinators remained neutral and by explicitly assuring patients that their responses would have no bearing on their medical treatment, the presence of clinical staff may still have influenced some participants to underreport negative behaviors. In addition, we must acknowledge the unavoidable selection bias inherent in our eligibility criteria. By excluding patients with severe psychiatric disorders, active malignancies, or significant communication barriers, our sample likely represents a subset of patients with better functional status and cognitive capacity than the general hemodialysis population. Consequently, the reported levels of HSB may be overestimated, as the most vulnerable patients—who likely face the greatest barriers to help‐seeking—were not fully represented. Building on this assessment tool, future research should prioritize the design and testing of a “stepped‐care intervention model.” For patients scoring low on the “symptom detection” dimension, research could evaluate the efficacy of gamified symptom education programs to enhance body awareness. For those struggling with “decision‐making,” cognitive behavioral therapy (CBT) targeting health anxiety and fear of medical burden should be investigated. Furthermore, for the “context‐dependent” profile identified in our LPA, future studies should test system‐level “nudge” interventions, such as incorporating mandatory symptom reporting pop‐ups in dialysis machines or implementing nurse‐led “symptom rounds” during the first hour of dialysis, to overcome environmental barriers to disclosure.

Lastly, while LPA offers valuable insights into latent subgroups, longitudinal designs are essential to explore whether these profiles remain stable over time or shift in response to clinical outcomes (e.g., hospitalization rates, quality of life). Such data could refine intervention timing—for example, identifying “at‐risk” transitions from context‐dependent to constrained profiles—and inform predictive models for preemptive care. Combining the HSB‐HD scale with biomarkers or wearable device data may further enhance its utility, bridging self‐reported behaviors with objective physiological indicators to create a holistic symptom management paradigm.

## 6. Conclusion

The development of the HSB‐HD scale provides a scientifically validated tool for assessing and managing the HSB of hemodialysis patients. The use of this scale is expected to improve the effectiveness of symptom management, thereby enhancing patients’ quality of life. With further extensive validation and application in the future, this scale is likely to become an important tool for improving symptom management in patients with CKD.

## Author Contributions

Xutong Zheng takes charge of conceptualization, data curation, formal analysis, methodology, project administration, software, supervision, validation, visualization, and roles/writing–original draft.

Aiping Wang takes charge of conceptualization, review, and editing.

## Funding

The authors received no specific funding for this work.

## Disclosure

This study has no patient or public involvement.

## Ethics Statement

The authors have nothing to report.

## Consent

The authors have nothing to report.

## Conflicts of Interest

The authors declare no conflicts of interest.

## Supporting Information

Additional supporting information can be found online in the Supporting Information section.

## Supporting information


**Supporting Information 1** Supporting File 1 revision of the scale.docx: This file presented details of the whole revision process of the scale.

**Supporting Information 2** Supporting File 2 content validity.docx: This file showed details of content analysis results appraised by each expert.

**Supporting Information 3** Supporting File 3 the full edition of the scale.docx: This file presented the final edition of the scale.

## Data Availability

The data that support the findings of this study are available from the corresponding author upon reasonable request.
