# Supplementary material for: Enhancing Patient‐Centered Communication in Hemodialysis Symptom Management Care‐Development and Validation of the HSB‐HD Scale for Assessing Help‐Seeking Behavior in Hemodialysis Patients: A Multiphase Cross‐Sectional Study
Source: J Nurs Manag. 2026 Feb 27;2026:8834451. doi: 10.1155/jonm/8834451 (PMC12947112; doi:10.1155/jonm/8834451)
Supplement: Supplementary file 1 — Supporting Information 1 Supporting File 1 revision of the scale.docx: This file presented details of the whole revision process of the scale. [file JONM-2026-8834451-s001.docx]

# Supplementary file 1 revision of the scale

**Process and details of item generation and revision**

| Dimension of the scale | The final edition of the item | Origin of the item | | | Details for revision |
| --- | --- | --- | --- | --- | --- |
|  |  | LR^†^ | EM | CI |  |
| Symptom Detection | 1. [I can notice new symptoms during hemodialysis.]我能够注意到血液透析过程中出现的新症状。 | ✔^‡^ |  |  |  |
|  | 2. [I can usually accurately perceive the discomfort caused by symptoms.] 我通常能够准确感知到症状造成的不适感。 |  | ✔ | ▲^‡^ | This item was proposed by an expert (E3) at expert meeting and originally read "I can usually accurately identify the discomfort associated with hemodialysis." The patient (P4) felt that the word "recognize" was a misnomer because it was too obscure to understand, so we changed it to " perceive." |
|  | 3. [I record every time a new or worsening symptom occurs.]每当有出现的新症状或加重的症状时我都会记录。 | **✔** |  |  |  |
|  | 4. [I can tell the changes of certain symptoms after dialysis.]我能够判断在透析后某些症状的变化。 |  | ✔ |  |  |
|  | 5. [When I feel more fatigued than usual after dialysis, I can usually notice the change.]当我透析后感觉比平时更加疲劳时，我通常能够注意到这一变化。 |  | ✔ | ▲ | This item was proposed by two experts (E2, E4) at the expert meeting, and the original content of this article was "I can judge the degree of change in symptoms after dialysis." After the cognitive interview, the patient felt that it needed to be modified. The patient (P10) felt that the subtle feeling of "symptom change" needed to be explained with examples, otherwise it was difficult to understand. The patient cited the symptom fatigue that he often experienced, and combined with the contents of the cognitive interview, we made modifications. |
| Symptom Interpretation | 6. [I am able to determine if some of my symptoms of discomfort require medical intervention.]我能够判断我的某些不适的症状是否需要医疗介入。 | ✔ |  |  |  |
|  | 7. [When I experience discomfort due to a symptom, I am usually able to identify the cause of the discomfort.]当我经历某种症状引起的不适时，我通常能够确定引起这种不适的原因。 | ✔ | ▲ |  | This item was taken from the literature and originally read, "When I feel that my body is experiencing some kind of discomfort, I can usually determine that dialysis is causing it." After expert discussion (E2, E3, E4), it is felt that there are many symptoms of end-stage renal disease patients, and not necessarily caused by hemodialysis, it may be caused by the disease itself or other treatments (such as taking drugs), so it is modified. |
|  | 8. [I know exactly what common symptoms hemodialysis can cause.]我清楚地知道血液透析可能导致哪些常见症状。 |  | ●^‡^ | ✔ |  |
|  | 9. [I know exactly what the common symptoms of chronic kidney disease can cause.]我清楚地知道慢性肾脏病可能导致哪些常见症状。 |  | ● | ✔ |  |
| Decision-Making for Help-Seeking | 10. [In the event of an exacerbation of suspected symptoms, I usually make a quick decision to seek medical help.]在出现疑似症状加重的情况下，我通常会迅速决定求助医护人员。 |  | ✔ | ▲ | This item was proposed by two experts (E4, E6) at expert meeting, and originally read “In the event of an exacerbation of suspected symptoms, I usually make a decision to seek medical help”. After cognitive interviews, patients said that they most often thought of seeking help when their symptoms worsened, but it was important to be able to respond immediately and quickly. It is therefore suggested that the word "quick" be added. |
|  | 11. [When I experience discomfort due to certain symptoms, I can tell if it's serious enough to warrant medical intervention.]当我经历某种症状引起的不适时，我能知道这是否足够严重需要医疗干预。 |  | ✔ |  |  |
|  | 12.[I can tell when a certain symptom of mine is a normal disease or reaction to hemodialysis] 我能够区分何时我的某个症状是正常的疾病或透析反应 | ✔ |  |  |  |
| timely disclosure and taking action | 13.[For dialysis-related discomfort, I usually consider when I need to seek professional medical help.]对于透析相关的不适，我通常会考虑何时需要寻求专业医疗帮助。 |  | ✔ | ▲ | This item was proposed by two experts (E3, E4) and originally read “For dialysis-related discomfort, I usually consider I need to seek professional medical help”. Through the cognitive interview with the patient, the patient (P5, P8) felt that the content of consideration was not clear enough. P8 pointed that it should be the time to consider seeking help, because some symptoms are mild at some times, do not need medical help, and can be solved by themselves. The important thing is to recognize when medical help is needed. |
|  | 14. [If I feel seriously unwell because of a symptom, I take immediate action to get help.]如果我因为某个症状感到严重不适，我会立即采取行动寻求帮助。 | ✔ |  |  |  |
|  | 15. [Once I decide to seek help, I will contact my doctor and the nurse in charge.]一旦决定寻求帮助，我会去联系我的主管医生和责任护士。 | ✔ | ▲ |  | This item was taken from the literature and originally read, “Once I decide to seek help, I will contact my doctor in charge”. At the expert meeting, the two experts (E2, E3) proposed that symptom management of hemodialysis is a multidisciplinary process and is mainly communicated and coordinated by doctors and nurses directly with patients rather than just doctors. Therefore, the role and status of nurses need to be emphasized. |
|  | 16.[When my symptoms worsen, I am able to seek immediate help from the medical team.] 当我的症状加重时，我能立即寻求医护团队的帮助。 | ✔ | ▲ |  | This item was taken from the literature and originally read, “When my symptoms worsen, I am able to seek immediate help from my doctor in charge”. At the expert meeting, the two experts (E2, E3) proposed that symptom management of hemodialysis is a multidisciplinary process and need to emphasize the significance of the team in symptom management. |
|  | 17.[I will promptly report any symptoms I experience to the medical staff.]  我会及时向医护人员报告我遇到的任何症状。 | ✔ | ▲ |  |  |
|  | 18.[I encourage family or friends to help me contact a doctor if they notice that I am unwell due to my symptoms.] 我会在家人或朋友发现我因症状产生的不适时鼓励他们帮助我联系医生。 |  | ● | ✔ |  |
|  | 19.[When I experience new symptoms related to my illness, I keep a detailed record for the next discussion with my doctor.] 当我经历疾病造成相关的新症状时，我会详细记录以便下次与医生讨论。 |  | ✔ | ▲ | This item was taken from the literature and originally read “When I experience new symptoms related to my illness, I keep a detailed record”. In the cognitive interview with the patient, the patient (P2, P4) stated that the purpose of the recording was to discuss the treatment of symptoms in detail with the doctor. So it's important to emphasize " for the next discussion with my doctor." |

**†** LR=literature review, EM= expert meeting, CI=cognitive interview.

**‡** ‘✔’ means the item was generated from this source, ‘▲’ means the item was revised by this source, ‘●’ means the item generated by the patients interview was reviewed by the expert.

**Demographic characteristics of the participants (patients)**

| Identification code | gender | age | education level | average income level (Yuan per month) | Time starting dialysis | dialysis times per week | Number of dialysis complications | Number of concurrent other chronic diseases | number of symptoms |
| --- | --- | --- | --- | --- | --- | --- | --- | --- | --- |
| P1 | male | 61 | primary school | ＜3000 | 1-5 years | 3 times per week | 2 | 1 | 1 |
| P2 | female | 65 | senior high school | 4000-4999 | ＞10 years | 3 times per week | 1 | 1 | 1 |
| P3 | male | 70 | primary school | 3000-3999 | ＜1 year | 3 times per week | 3 | 2 | 3 |
| P4 | female | 43 | undergraduate | ＜3000 | 1-5 years | 3 times per week | 1 | 1 | 3 |
| P5 | male | 55 | primary school | ＜3000 | 6-10 years | 3 times per week | 1 | 1 | 5 |
| P6 | female | 64 | senior high school | ＜3000 | ＜1 year | 3 times per week | 4 | 1 | 5 |
| P7 | female | 51 | junior college | 3000-3999 | 1-5 years | 3 times per week | 1 | 1 | 9 |
| P8 | male | 37 | undergraduate | ≥5000 | 1-5 years | 1 time per week | 1 | 1 | 9 |
| P9 | female | 68 | primary school | 3000-3999 | 1-5 years | 1 time per week | 5 | 6 | 22 |
| P10 | male | 39 | junior college | ＜3000 | 1-5 years | 3 times per week | 4 | 3 | 25 |

**Demographic characteristics of the experts**

| Identification code | Research field | Gender | age | Years of experience in the profession |
| --- | --- | --- | --- | --- |
| E1 | Epidemiology and statistics | Male | 58 | 33 |
| E2 | Nursing (Chronic disease management) | Female | 57 | 37 |
| E3 | Nursing (self-management of Chronic Kidney Disease) | Female | 46 | 25 |
| E4 | Nephrology | Female | 48 | 21 |
| E5 | Behavioral medicine | Male | 55 | 26 |
| E6 | Psychology | Female | 42 | 23 |
