# Supplementary material for: Enhancing Patient‐Centered Communication in Hemodialysis Symptom Management Care‐Development and Validation of the HSB‐HD Scale for Assessing Help‐Seeking Behavior in Hemodialysis Patients: A Multiphase Cross‐Sectional Study
Source: J Nurs Manag. 2026 Feb 27;2026:8834451. doi: 10.1155/jonm/8834451 (PMC12947112; doi:10.1155/jonm/8834451)
Supplement: Supplementary file 2 — Supporting Information 2 Supporting File 2 content validity.docx: This file showed details of content analysis results appraised by each expert. [file JONM-2026-8834451-s002.docx]

| Item number | Expert code | | | | | | | I-CVI |
| --- | --- | --- | --- | --- | --- | --- | --- | --- |
|  | A | B | C | D | E | F | G |  |
| 1 | 4 | 4 | 4 | 4 | 4 | 3 | 4 | 1 |
| 2 | 4 | 3 | 4 | 3 | 4 | 4 | 3 | 1 |
| 3 | 4 | 3 | 4 | 4 | 4 | 4 | 4 | 1 |
| 4 | 4 | 4 | 3 | 3 | 4 | 2 | 4 | 0.86 |
| 5 | 4 | 4 | 4 | 4 | 4 | 4 | 4 | 1 |
| 6 | 4 | 4 | 4 | 4 | 4 | 4 | 4 | 1 |
| 7 | 4 | 3 | 4 | 4 | 4 | 4 | 4 | 1 |
| 8 | 4 | 3 | 4 | 4 | 4 | 4 | 4 | 1 |
| 9 | 4 | 4 | 3 | 3 | 4 | 2 | 4 | 0.86 |
| 10 | 4 | 3 | 4 | 4 | 4 | 4 | 4 | 1 |
| 11 | 4 | 4 | 4 | 4 | 4 | 4 | 4 | 1 |
| 12 | 4 | 4 | 4 | 4 | 4 | 2 | 4 | 0.86 |
| 13 | 4 | 4 | 4 | 4 | 4 | 4 | 4 | 1 |
| 14 | 4 | 4 | 4 | 4 | 4 | 1 | 4 | 0.86 |
| 15 | 4 | 3 | 4 | 3 | 4 | 4 | 4 | 1 |
| 16 | 4 | 4 | 4 | 4 | 4 | 4 | 4 | 1 |
| 17 | 3 | 4 | 4 | 4 | 4 | 2 | 4 | 0.86 |

# Supplementary File 2 content validity
