# Supplementary material for: Enhancing Patient‐Centered Communication in Hemodialysis Symptom Management Care‐Development and Validation of the HSB‐HD Scale for Assessing Help‐Seeking Behavior in Hemodialysis Patients: A Multiphase Cross‐Sectional Study
Source: J Nurs Manag. 2026 Feb 27;2026:8834451. doi: 10.1155/jonm/8834451 (PMC12947112; doi:10.1155/jonm/8834451)
Supplement: Supplementary file 3 — Supporting Information 3 Supporting File 3 the full edition of the scale.docx: This file presented the final edition of the scale. [file JONM-2026-8834451-s003.docx]

# Supplementary File 3 the full edition of the scale

| Dimension of the scale | The final edition of the item |
| --- | --- |
|  |  |
| Symptom Detection | 1. [I can notice new symptoms during hemodialysis.]我能够注意到血液透析过程中出现的新症状。 |
|  | 2. [I can usually accurately perceive the discomfort caused by symptoms.] 我通常能够准确感知到症状造成的不适感。 |
|  | 3. [I record every time a new or worsening symptom occurs.]每当有出现的新症状或加重的症状时我都会记录。 |
|  | 4. [I can tell the changes of certain symptoms after dialysis.]我能够判断在透析后某些症状的变化。 |
| Symptom Interpretation | 5. [I am able to determine if some of my symptoms of discomfort require medical intervention.]我能够判断我的某些不适的症状是否需要医疗介入。 |
|  | 6. [When I experience discomfort due to a symptom, I am usually able to identify the cause of the discomfort.]当我经历某种症状引起的不适时，我通常能够确定引起这种不适的原因。 |
|  | 7. [I know exactly what common symptoms hemodialysis can cause.]我清楚地知道血液透析可能导致哪些常见症状。 |
|  | 8. [I know exactly what the common symptoms of chronic kidney disease can cause.]我清楚地知道慢性肾脏病可能导致哪些常见症状。 |
|  | 9. [When I experience discomfort due to certain symptoms, I can tell if it's serious enough to warrant medical intervention.]当我经历某种症状引起的不适时，我能知道这是否足够严重需要医疗干预。 |
|  | 10.[I can tell when a certain symptom of mine is a normal disease or reaction to hemodialysis] 我能够区分何时我的某个症状是正常的疾病或透析反应 |
| timely disclosure and taking action | 11.[For dialysis-related discomfort, I usually consider when I need to seek professional medical help.]对于透析相关的不适，我通常会考虑何时需要寻求专业医疗帮助。 |
|  | 12. [If I feel seriously unwell because of a symptom, I take immediate action to get help.]如果我因为某个症状感到严重不适，我会立即采取行动寻求帮助。 |
|  | 13. [Once I decide to seek help, I will contact my doctor and the nurse in charge.]一旦决定寻求帮助，我会去联系我的主管医生和责任护士。 |
|  | 14.[When my symptoms worsen, I am able to seek immediate help from the medical team.] 当我的症状加重时，我能立即寻求医护团队的帮助。 |
|  | 15.[I will promptly report any symptoms I experience to the medical staff.]  我会及时向医护人员报告我遇到的任何症状。 |
|  | 16.[I encourage family or friends to help me contact a doctor if they notice that I am unwell due to my symptoms.] 我会在家人或朋友发现我因症状产生的不适时鼓励他们帮助我联系医生。 |
|  | 17.[When I experience new symptoms related to my illness, I keep a detailed record for the next discussion with my doctor.] 当我经历疾病造成相关的新症状时，我会详细记录以便下次与医生讨论。 |
